# Predisposing Factors and Neurologic Outcomes of Patients with Elevated Serum Amylase and/or Lipase after Out-of-Hospital Cardiac Arrest: A Retrospective Cohort Study

**DOI:** 10.3390/jcm11051426

**Published:** 2022-03-04

**Authors:** Shin Young Park, Min Joung Kim, Incheol Park, Ha Yan Kim, Myeongjee Lee, Yoo Seok Park, Sung Phil Chung

**Affiliations:** 1 Department of Emergency Medicine, Yonsei University College of Medicine, 50 Yonsei-ro, Seodaemun-gu, Seoul 03722, Korea; chilli89@yuhs.ac (S.Y.P.); boringzzz@yuhs.ac (M.J.K.); incheol@yuhs.ac (I.P.); emstar@yuhs.ac (S.P.C.); 2 Biostatistics Collaboration Unit, Department of Biomedical Systems Informatics, Yonsei University College of Medicine, 50 Yonsei-ro, Seodaemun-gu, Seoul 03722, Korea; hykim1213@yuhs.ac (H.Y.K.); mlee1004@yuhs.ac (M.L.)

**Keywords:** elevated serum pancreatic enzyme levels, out-of-hospital cardiac arrest, post-cardiac arrest syndrome, neurologic outcome, mortality

## Abstract

This study investigated the patient outcomes, incidence, and predisposing factors of elevated pancreatic enzyme levels after OHCA. We conducted a retrospective cohort study of patients treated with targeted temperature management (TTM) after out-of-hospital cardiac arrest (OHCA). Elevation of pancreatic enzyme levels was defined as serum amylase or lipase levels that were at least three times the upper limit of normal. The factors associated with elevated pancreatic enzyme levels and their association with neurologic outcomes and mortality 28 days after OHCA were analyzed. Among the 355 patients, 166 (46.8%) patients developed elevated pancreatic enzyme levels. In the multivariable analysis (odds ratio, 95% confidence interval), initial shockable rhythm (0.62, 0.39–0.98, *p* = 0.04), time from collapse to return of spontaneous circulation (1.02, 1.01–1.04, *p* < 0.001), and history of coronary artery disease (1.7, 1.01–2.87, *p* = 0.046) were associated with elevated pancreatic enzyme levels. After adjusting for confounding factors, elevated pancreatic enzyme levels were associated with neurologic outcomes (5.44, 3.35–8.83, *p* < 0.001) and mortality (3.74, 2.39–5.86, *p* < 0.001). Increased pancreatic enzyme levels are common in patients treated with TTM after OHCA and are associated with unfavorable neurologic outcomes and mortality at 28 days after OHCA.

## 1. Introduction

Despite significant advances in post-cardiac arrest care [1], hospital mortality remains high in patients who experience out-of-hospital cardiac arrest (OHCA) [2,3]. Post-cardiac arrest syndrome is defined by unique and complex combinations of pathophysiological processes after the return of spontaneous circulation (ROSC). It comprises post-cardiac arrest brain injury, myocardial dysfunction, systemic ischemia/reperfusion response, and persistent precipitating pathology [4]. Of these, systemic ischemia/reperfusion response may be compared to a sepsis-like syndrome, which causes multiple organ damage [5]. Although many studies on solid organ damage have evaluated the kidney and liver, only a few studies have investigated pancreatic damage after ROSC [6,7,8,9,10].

The clinical course of acute pancreatitis ranges from recovery without special treatment to death [11]. Despite significant progress in the treatment of acute pancreatitis, morbidity and mortality rates remain high [12]. An animal study reported that ischemia/reperfusion injury of the pancreas caused acute pancreatitis [13]. Moreover, blood flow suppression (total or near-total) by cross-clamping of the descending thoracic aorta during aortic aneurysm surgery, even for a brief duration, resulted in significant pancreatic injury [14]. The increase in serum pancreatic enzyme level was also correlated with the aortic clamping time. However, there are only a few studies on ischemic pancreatitis in association with cardiac arrest. Polderman [15] reported that hypothermia treatment in post-cardiac arrest syndrome often resulted in elevated levels of amylase in the blood, but the risk of clinically severe pancreatitis was low. However, Choi et al. [16] reported that acute pancreatitis was the most common complication after hypothermia treatment in patients with ROSC after cardiac arrest due to drowning. Moreover, to the best of our knowledge, no study in the literature has evaluated the relationship between increased pancreatic enzyme levels and neurologic prognosis in patients with OHCA after targeted temperature management (TTM). Therefore, this study aimed to investigate the incidence of and predisposing factors for elevated serum amylase and/or lipase after OHCA, as well as patient outcomes.

## 2. Materials and Methods

### 2.1. Study Design and Population

We performed a retrospective analysis of prospectively collected data using the registry of a critical pathway (CP) for post-resuscitation care, including TTM, from Yonsei University College of Medicine, Severance Hospital, Seoul, Republic of Korea, from September 2011 to October 2020. Patients who recovered spontaneous circulation after OHCA were selected for CP activation according to the inclusion and exclusion criteria [17]. We collected data of the index cardiac arrest event and outcomes according to the Utstein Style recommendation for reporting cardiac arrest research [18]. Post-cardiac arrest care, including TTM, was conducted in accordance with international guidelines. Patients aged <19 years, those who died within 24 h after ROSC, those transferred to another hospital, those whose families demanded for withdrawal of life-sustaining treatment during TTM, those with cardiac arrest due to trauma to exclude traumatic pancreatic damage, those diagnosed with pancreatitis before cardiac arrest, those diagnosed with pancreatic and biliary cancer, and those without serum amylase and lipase level data during the first 24 h after ROSC were excluded from the analysis. The study was reviewed and approved by the Institutional Review Board of Yonsei University College of Medicine, Severance Hospital (approval reference No. 4-2021-0567). The requirement for informed consent was waived by the ethics committee because of the retrospective nature of the study.

### 2.2. Data Collection

The patients’ demographic data (age, sex, weight, height, and comorbidities), resuscitation variables (initial rhythm, witnessed or unwitnessed arrest, bystander cardiopulmonary resuscitation (CPR) status, time from collapse to ROSC, presence or absence of defibrillation, total dose of epinephrine, and cause of arrest) were collected. Based on the CP protocol of our institution, serum amylase and lipase levels were measured immediately, 12 h, and 24 h after ROSC. However, the attending physician decided whether to perform additional tests for patients with elevated pancreatic enzyme levels. Based on previous studies that a significant increase in serum pancreatic enzyme levels peaks approximately 24 h after ischemic injury to the pancreas [14], we collected serum amylase and lipase data for up to 24 h in this study. The use of vasoactive agents, extracorporeal membrane oxygenation, and continuous renal replacement therapy (CRRT) were also recorded. Post-cardiac arrest shock was defined as the need for vasopressors or inotropics to maintain a mean arterial pressure of >65 mmHg even after fluid administration within 72 h of cardiac arrest.

### 2.3. Definition of Elevated Serum Pancreatic Enzyme Level

Acute pancreatitis was said to occur if more than two of the following three features were present: (1) typical abdominal pain of acute pancreatitis (acute upper abdominal pain radiating to the back), (2) serum amylase (normal value < 115 U/L) or lipase (normal value < 60 U/L) levels at least three times the upper limit of normal, and (3) characteristic findings of acute pancreatitis on computed tomography (CT) and, less commonly, magnetic resonance imaging or transabdominal ultrasonography [19,20,21,22,23]. However, abdominal pain cannot be evaluated in patients who receive TTM after cardiac arrest and an abdominal imaging study such as CT is not usually performed after cardiac arrest. Therefore, in this study, elevation of serum pancreatic enzyme levels was defined as a serum amylase or lipase level that was at least three times the upper limit of normal within 24 h after ROSC. Additionally, we divided elevated serum pancreatic enzyme levels into three groups: elevated amylase, elevated lipase, and elevated amylase and lipase levels.

### 2.4. Outcome Measures

The primary outcome of this study was the neurologic outcome at 28 days after ROSC. The Cerebral Performance Category score (CPC 1 = conscious and alert with good cerebral performance, CPC 2 = moderate cerebral disability, CPC 3 = severe cerebral disability, CPC 4 = coma or vegetative state, and CPC 5 = brain death) was used to assess neurologic outcome, with a CPC score of 1–2 being regarded as a favorable neurologic outcome and a score of 3–5 as an unfavorable neurologic outcome [24]. The secondary outcome was 28-day mortality.

### 2.5. Statistical Methods

Statistical analysis was performed using SAS (version 9.4; SAS Inc., Cary, NC, USA) and the R package ‘rms’ (version 3.6.3, http://www.R-project.org). Categorical variables are presented as frequency (%), whereas continuous variables are presented as mean ± standard deviation. The independent two-sample *t*-test was used to compare continuous variables, and the chi-square test or Fisher’s exact test was used to compare categorical variables, as appropriate. Changes in serum amylase and lipase levels over time were analyzed using a linear mixed model in patients with elevated pancreatic enzyme levels. For the evaluation of the risk factors associated with elevated serum pancreatic enzyme levels, univariate logistic regression analysis was performed using the demographic and resuscitation variables. Thereafter, multivariable logistic regression analysis was performed with variables that had a *p*-value of <0.1 in the univariate logistic regression. To assess the association between elevated pancreatic enzyme levels and neurologic outcomes or mortality, we conducted multivariable logistic regression analysis after covariate adjustment. We conducted subgroup analysis according to the division of elevated serum pancreatic enzyme levels. To assess adjusted relationships between the maximum pancreatic enzyme level within 24 h and prognosis, we used restricted cubic spline regression models with 4 knots placed at the 5th, 35th, 65th, and 95th percentiles. The maximum serum amylase or lipase level was employed in the logistic regression analysis with the spline term after covariate adjustment. All reported *p*-values are two-sided, and statistical significance was set at *p* < 0.05.

## 3. Results

### 3.1. Baseline Characteristics

**Figure 1:**
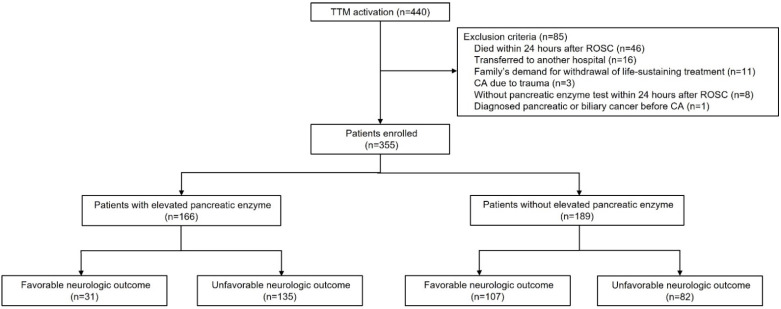
Flow diagram of the study. Abbreviations: TTM, targeted temperature management; ROSC, return of spontaneous circulation; CA, cardiac arrest.

From September 2011 to October 2020, 440 patients with ROSC after OHCA underwent TTM. Of these, we excluded 85 patients based on the set criteria. Overall, 355 patients were enrolled in this study (Figure 1). Details of the baseline characteristics of the study population are shown in Table 1.

### 3.2. Characteristics of and Risk Factors for Elevated Pancreatic Enzyme Levels

**Figure 2:**
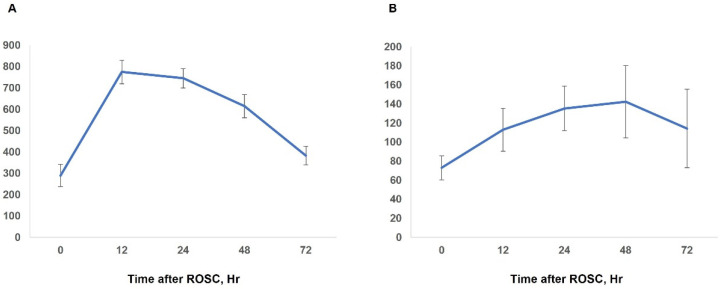
Trends in serum amylase and lipase levels in patients with elevated pancreatic enzyme levels. Estimated means and standard errors obtained by linear mixed model are plotted on the y-axes. The solid lines represent the estimated means with vertical lines denoting the standard errors. Serum amylase (U/L) (**A**) and lipase (U/L) levels (**B**).

Increased pancreatic enzyme levels within 24 h after ROSC occurred in 166 patients (46.8%). The mean age of patients with elevated pancreatic enzyme levels (65.0 ± 15.6 years) was significantly higher than that of patients without elevated pancreatic enzyme levels (61.5 ± 15.5 years). Among the laboratory test results, the amylase level (775.2 ± 691.9 U/L) measured at 12 h after ROSC was the highest, while lipase level (135.3 ± 292.7 U/L) was the highest after 24 h in patients with elevated pancreatic enzyme levels. In those patients, serum amylase and lipase tests were performed in 133 patients at 48 h after ROSC and in 85 patients at 72 h after ROSC. The amylase levels measured at 48 and 72 h after ROSC were 615.2 ± 574.8 U/L and 383.5 ± 401.2 U/L, respectively. These levels were lower than those measured at 12 h and 24 h (all *p* < 0.001). The lipase levels measured at 48 and 72 h after ROSC were 142.3 ± 401.4 U/L and 114.3 ± 379.3 U/L, respectively. However, the lipase level measured at 48 h was similar to that measured at 12 h and 24 h (*p* = 0.46 and *p* = 0.92, respectively) (Figure 2). Moreover, only six patients underwent an abdominal CT after increased pancreatic enzyme levels were noted. Among them, findings of pancreatitis were found in three patients. The initial shockable rhythm was 30.7% in the group with elevated pancreatic enzyme levels and 46.0% in patients without elevated pancreatic enzyme levels, and the difference was significant (*p* = 0.003). The mean time from collapse to ROSC for the group with elevated pancreatic enzyme levels was 33.9 ± 21.0 min, which was significantly longer than that for the group without elevated pancreatic enzyme level (25.1 ± 19.3 min, *p* < 0.001). In the multivariate analysis, initial shockable rhythm (odds ratio [OR], 0.62; 95% confidence interval [CI], 0.39–0.98; *p* = 0.04], time from collapse to ROSC (OR, 1.02; 95% CI, 1.01–1.04; *p* < 0.001), and medical history of coronary artery disease (OR, 1.7; 95% CI, 1.01–2.87; *p* = 0.046) were associated with elevated pancreatic enzyme levels (Table 2).

### 3.3. Neurologic Outcome and 28-Day Mortality

We observed significantly more unfavorable neurologic outcomes and higher 28-day mortality rates in patients with elevated pancreatic enzyme levels after OHCA than in patients without elevated pancreatic enzyme levels. The rates of unfavorable neurologic outcomes and 28-day mortality were 81.3% and 56.0%, respectively, in patients with elevated pancreatic enzyme levels compared to 42.4% and 25.4%, respectively, in patients without elevated pancreatic enzyme levels (both *p* < 0.001).

Factors associated with neurologic outcomes and 28-day mortality are described in Table 3. A total of 219 patients (61.7%) had unfavorable neurologic outcomes at 28 days after ROSC. In the univariate logistic regression analysis, patient age, sex, witnessed arrest, bystander CPR, shockable rhythm, cause of arrest, time from collapse to ROSC, medical history (such as diabetes mellitus and renal disease), post-cardiac arrest shock, CRRT application, and increase in pancreatic enzyme levels were significantly associated with neurologic prognosis. Of these, male sex (OR, 2.96; 95% CI, 1.20–7.28; *p* = 0.02), bystander CPR (OR, 0.39; 95% CI, 0.19–0.81; *p* = 0.01), shockable rhythm (OR, 0.44; 95% C,: 0.22–0.85; *p* = 0.012), time from collapse to ROSC (per minute) (OR, 1.05; 95% CI, 1.04–1.07; *p* < 0.001), cardiac cause of arrest (OR, 0.26; 95% CI, 0.12–0.53; *p* < 0.001), CRRT application (OR, 3.77; 95% CI, 1.22–11.65; *p* = 0.02), and elevated pancreatic enzyme levels (OR, 5.44; 95% CI, 3.35–8.83; *p* < 0.001) were associated with unfavorable neurologic outcomes as determined by multivariable logistic regression analysis (Table 4). At 28 days, 141 patients (39.7%) had died, and elevated pancreatic enzyme levels were also associated with 28-day mortality in the multivariable logistic regression analysis (OR, 3.74; 95% CI, 0.39–5.86; *p* < 0.001).

The subgroup analysis of elevated pancreatic enzyme levels compared with nonelevated pancreatic enzyme levels showed that amylase elevation alone was associated with both unfavorable neurologic outcomes (OR, 6.03; 95% CI; 3.03–12.00; *p* < 0.001) and 28-day mortality (OR, 2.8; 95% CI; 1.58–4.93; *p* < 0.001). Elevation of both amylase and lipase levels was only associated with unfavorable neurologic outcomes (OR, 7.08; 95% CI, 1.73–29.02; *p* < 0.01). However, lipase elevation alone was not associated with unfavorable neurologic outcomes or 28-day mortality.

**Figure 3:**
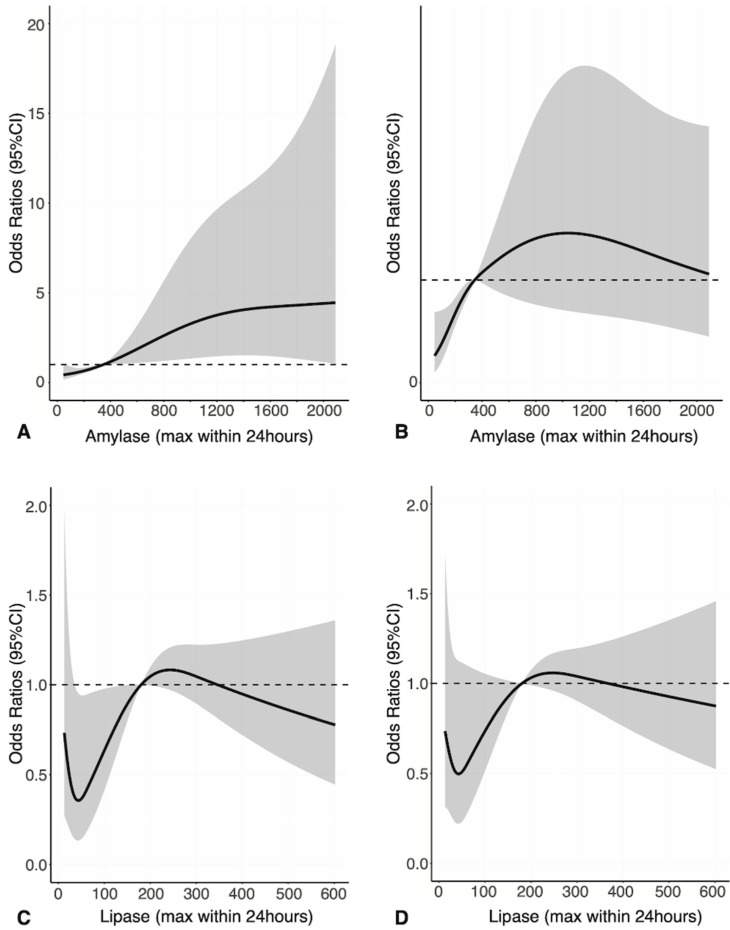
Adjusted relationships between the maximum serum amylase or lipase levels within 24 h and neurologic outcome or 28-day mortality. Estimated adjusted odds ratios (ORs) and 95% confidence intervals (CIs) obtained by multivariable logistic regression using restricted cubic splines are plotted on the y-axes. The solid lines represent the estimated adjusted ORs, with shaded ribbons denoting the 95% CIs. The horizontal dotted lines represent the OR of 1.0, which indicates the OR of maximum serum amylase at 345 U/L or lipase at 180 U/L. Relationships between maximum serum amylase and (**A**) neurologic outcome or (**B**) 28-day mortality and relationships between maximum lipase and (**C**) neurologic outcome or (**D**) 28-day mortality. Abbreviations: CI, confidence interval.

In patients with unfavorable neurologic outcome, the maximum serum amylase within 24 h was 629.5 ± 730.5 U/L compared to 258.7 ± 297.5 U/L in patients with favorable neurologic outcomes (*p* < 0.001). To assess the relationship of maximum serum amylase and lipase levels with prognosis, we used restricted cubic spline regression models. When a reference amylase level of 345 U/L (three times the upper limit of normal) was used, the higher the maximum serum amylase level, the higher the odds for an unfavorable neurologic outcome (Figure 3A). However, there was no association between maximum serum amylase and 28-day mortality and between maximum serum lipase and neurologic outcomes or mortality (Figure 3B–D).

## 4. Discussion

In our study, of 355 patients who underwent TTM after OHCA, the prevalence of elevated pancreatic enzyme was 47%. We also observed that increase in pancreatic enzyme levels was associated with an unfavorable neurologic outcome and 28-day mortality. Furthermore, initial rhythm, time from collapse to ROSC, and history of coronary artery disease were factors associated with the development of elevated pancreatic enzyme levels. This study is the first to investigate the incidence, neurologic outcome, and predisposing factors of elevated pancreatic enzyme levels in patients treated with TTM after OHCA.

Systemic ischemia/reperfusion response, which is similar to sepsis syndrome [25], is an important component of post-cardiac arrest syndrome and causes multiple organ damage. Many studies on organ damage after cardiac arrest have involved the cardiovascular, respiratory, renal, and hepatic systems [6,7,8,9,10], but, to the best of our knowledge, there has been no such study on pancreatic damage after ROSC. The pancreas is sensitive to hypoxia and ischemia [14,26]. The role of ischemia in pancreatitis has been well reported in both clinical and laboratory studies [27,28,29]. However, we believe that there is only one case report about hypoxic pancreatitis following cardiac arrest, and in this case, the patient survived but developed ischemic encephalopathy [30]. In the present study, increase in pancreatic enzyme levels was a frequent complication and was related to an unfavorable neurologic prognosis and mortality in patients treated with TTM after OHCA.

Many studies have reported the mortality rate of acute pancreatitis [31,32,33,34]. The mortality rate among patients with acute pancreatitis is reported to be relatively high (21%), and the severity of the disease is a major factor affecting the mortality rate [31]. Our study also showed that patients with TTM after OHCA who developed increased pancreatic enzyme levels had unfavorable neurologic outcomes (81.3%) and a high mortality rate (56.0%). However, it is unclear whether this was due to elevated pancreatic enzyme levels or other causes such as hypoxic–ischemic encephalopathy.

Serum amylase and lipase levels are commonly used as laboratory tests for diagnosing acute pancreatitis, but there are few studies about the effect of their levels on prognosis. A previous study showed that serum amylase and/or lipase levels decreased with worsening chronic pancreatitis [35], and another study in patients with acute paraquat poisoning reported that the higher the amylase level, the higher the mortality rate [36]. Patients with elevated pancreatic enzyme levels in the neurosurgery intensive care unit have a higher mortality rate than patients with normal enzyme levels [33]. In an animal study, hyperamylasemia was suggested but not confirmed as a pathogenic factor of pancreatic encephalopathy [37] and a possible contributor to the development of brain damage [38]. In our study, neurologic outcomes in patients with elevated pancreatic enzyme levels were worse when the amylase level was higher. However, above the reference amylase level (345 U/L), there was no difference in the 28-day mortality, even if the maximum serum amylase level increased further. Moreover, in the subgroup analysis, elevation of the amylase level alone was associated with an unfavorable neurologic outcome and 28-day mortality, but elevation of lipase level alone did not affect the prognosis. Further studies may be needed to determine the reason for such a result.

In this study, positive findings were found in only three of six patients who underwent abdominal CT after an increase in pancreatic enzyme levels. However, it is not yet known when the imaging changes of the pancreas will appear on CT in patients who have undergone cardiac arrest.

There are several limitations to this study. First, the generalizability of the results may be limited as the study was conducted in a single center. Second, we could not use the concept of acute pancreatitis due to hypoxic injury. Previously, the diagnosis was made when two or more of the three diagnostic criteria for acute pancreatitis were met, although a history of abdominal pain could not be recorded and abdominal CT is not usually performed in patients with OHCA. Therefore, in this study, only laboratory tests (amylase and lipase) were used. In addition, based on a previous study in which amylase and lipase levels peaked at 24 h, the laboratory tests were conducted for up to 24 h. Therefore, patients with elevated amylase or lipase levels 24 h after ROSC were not included in our study. Finally, some diseases affecting the serum amylase and/or lipase elevation, such as salivary gland lesions, renal failure, liver failure, peptic ulcer and biliary or gastrointestinal tract inflammation, and drugs that could cause amylase and/or lipase elevation were not considered [39,40]. Therefore, additional studies that can address these shortcomings are needed.

## 5. Conclusions

Increased serum pancreatic enzyme levels were found to be a common complication and a potent predictor of unfavorable neurologic outcomes and 28-day mortality in patients treated with TTM after OHCA. Initial rhythm, time from collapse to ROSC, and history of coronary artery disease were associated with elevated pancreatic enzyme levels.

## Figures and Tables

**Table 1:** Baseline characteristics, cardiac arrest-related information, and outcomes of patients with and without elevated serum amylase and/or lipase levels.

	All Patients	Non-Elevated Pancreatic Enzyme Levels	Elevated Pancreatic Enzyme Levels	*p*-Value
	(*n* = 355)	(*n* = 189, 53.2%)	(*n* = 166, 46.8%)	
**Patients’ demographic data**				
Age (years)	63.1 ± 15.6	61.5 ± 15.5	65.0 ± 15.6	0.03
Sex, male	266 (74.9)	139 (73.5)	127 (76.5)	0.52
Weight (kg)	64.9 ± 12.4	64.7 ± 13.1	65.1 ± 11.6	0.77
Height (cm)	166.4 ± 9.0	166.7 ± 10.5	166.1 ± 7.2	0.53
**CA information**				
Witnessed	255 (71.8)	142 (75.1)	113 (68.1)	0.14
Bystander CPR	248 (69.9)	139 (73.5)	109(65.7)	0.11
Shockable rhythm (VF/VT)	138 (38.9)	87 (46.0)	51 (30.7)	0.003
Cardiac cause	225 (63.4)	127 (67.2)	98 (59)	0.11
Time from collapse to ROSC (minutes)	29.2 ± 20.6	25.1 ± 19.3	33.9 ± 21.0	<0.001
Total dose of epinephrine (mg)	2.5 ± 3.2	2.1 ± 3.1	3.0 ± 3.3	0.01
**Comorbidities**				
Hypertension	135 (38)	70 (37)	65 (39.2)	0.68
Diabetes mellitus	90 (25.4)	46 (24.3)	44 (26.5)	0.64
Coronary artery disease	86 (24.2)	38 (20.1)	48 (28.9)	0.053
Cerebrovascular accident	28 (7.9)	10 (5.3)	18 (10.8)	0.053
Pulmonary disease	33 (9.3)	20 (10.6)	13 (7.8)	0.37
Renal disease	46 (13)	22 (11.6)	24 (14.5)	0.43
Liver cirrhosis	2 (0.6)	0	2 (1.2)	0.22
Other cancer	27 (7.7)	17 (9)	10 (6)	0.29
**Laboratory test levels (U/L)**				
Amylase at 0 h (*n* = 232)	179.9 ± 362.6	95.3 ± 49.7	289.6 ± 526.5	<0.001
Amylase at 12 h (*n* = 333)	422.1 ± 575.3	118.2 ± 71.4	775.2 ± 691.9	<0.001
Amylase at 24 h (*n* = 326)	417.3 ± 510.3	116.2 ± 77.9	745.4 ± 575.9	<0.001
Maximum amylase	487.4 ± 628.5	141.1 ± 80.2	881.7 ± 739.1	<0.001
Lipase at 0 h (*n* = 232)	55.9 ± 87.5	42.5 ± 22.0	73.1 ± 127.9	0.004
Lipase at 12 h (*n* = 331)	69.2 ± 194.7	31.1 ± 24.2	112.9 ± 278.3	<0.001
Lipase at 24 h (*n* = 328)	80.0 ± 209.8	29.2 ± 24.8	135.3 ± 292.7	<0.001
Maximum lipase	108.0 ± 242.9	46.1 ± 29.7	178.5 ± 340.9	<0.001
**Outcome**				
Unfavorable neurologic outcome	219 (61.7)	84 (44.4)	135 (81.3)	<0.001
28-day mortality	141 (39.7)	48 (25.4)	93 (56)	<0.001

Data are expressed as mean ± standard deviation or *n* (%). Abbreviations: CA, cardiac arrest; CPR, cardiopulmonary resuscitation; VF, ventricular fibrillation; VT, ventricular tachycardia; ROSC, return of spontaneous circulation.

**Table 2:** Multivariable logistic regression model for risk factors associated with elevated serum amylase and/or lipase levels.

	OR	95% CI	*p*-Value
Shockable rhythm (VF/VT)	0.62	0.39–0.98	0.04
Time from collapse to ROSC (per minute)	1.02	1.01–1.04	<0.001
Coronary artery disease	1.7	1.01–2.87	0.046

Abbreviations: OR, odds ratio; CI, confidence interval; VF, ventricular fibrillation; VT, ventricular tachycardia; ROSC, return of spontaneous circulation.

**Table 3:** Characteristics of the study population according to neurologic outcomes and 28-day mortality.

	Neurologic Outcome			28-Day Mortality		
	Favorable	Unfavorable	*p*-Value	Survival	Death	*p*-Value
	(*n* = 136, 38.3%)	(*n* = 219, 61.7%)		(*n* = 214, 60.3%)	(*n* = 141, 39.7%)	
**Patients’ demographic data**						
Age (years)	61.0 ± 14.9	64.5 ± 16.0	0.04	61.1 ± 15.8	66.2 ± 14.6	0.003
Sex, male	113 (83.1)	153 (69.9)	0.005	166 (77.6)	100 (70.9)	0.16
Weight (kg)	65.3 ± 13.4	64.7 ± 11.8	0.61	64.2 ± 13.1	65.9 ± 11.3	0.21
Height (cm)	167.9 ± 8.7	165.5 ± 9.2	0.01	167 ± 10.0	166 ± 7.4	0.43
**CA information**						
Witnessed	112 (82.4)	143 (65.3)	<0.001	170 (79.4)	85 (60.3)	<0.001
Bystander	114 (83.8)	134 (61.2)	<0.001	166 (77.6)	82 (58.2)	<0.001
Shockable rhythm (VF/VT)	82 (60.3)	56 (25.6)	<0.001	108 (50.5)	30 (21.3)	<0.001
Cardiac cause	108 (79.4)	117 (53.4)	<0.001	149 (69.6)	76 (53.9)	0.003
Time from collapse to ROSC (minutes)	18.8 ± 15.6	35.7 ± 20.7	<0.001	23 ± 18.4	38.5 ± 20.2	<0.001
Total dose of epinephrine (mg)	1.4 ± 2.7	3.2 ± 3.4	<0.001	1.8 ± 2.7	3.6 ± 3.7	<0.001
**Comorbidities**						
Hypertension	50 (36.8)	85 (38.8)	0.7	76 (35.5)	59 (41.8)	0.23
Diabetes mellitus	26 (19.1)	64 (29.2)	0.03	43 (20.1)	47 (33.3)	0.005
Coronary artery disease	38 (27.9)	48 (21.9)	0.2	53 (24.8)	33 (23.4)	0.77
Cerebrovascular accident	8 (5.9)	20 (9.1)	0.27	11 (5.1)	17 (12.1)	0.02
Pulmonary disease	9 (6.6)	24 (11.0)	0.17	20 (9.3)	13 (9.2)	0.97
Renal disease	6 (4.4)	40 (18.3)	<0.001	19 (8.9)	27 (19.1)	0.005
Liver cirrhosis	0	2 (0.9)	0.53	0	2 (1.4)	0.16
Other cancer	8 (5.9)	19 (8.7)	0.33	15 (7.0)	12 (8.5)	0.60
**Laboratory test levels (U/L)**						
Amylase at 0 h (*n* = 232)	106.8 ± 66.9	236.9 ± 473.0	0.001	120.6 ± 92.3	274.3 ± 560.8	<0.001
Amylase at 12 h (*n* = 333)	225.7 ± 295.8	544.6 ± 667.0	<0.001	328.3 ± 493.7	572.2 ± 660.9	0.001
Amylase at 24 h (*n* = 326)	224.9 ± 269.7	540.1 ± 585.0	<0.001	332.0 ± 482.6	554.4 ± 525.5	0.03
Maximum amylase	258.7 ± 297.5	629.5 ± 730.5	<0.001	375.8 ± 538.9	656.8 ± 713.4	0.001
Lipase at 0 h (*n* = 232)	45.0 ± 36.8	64.4 ± 111.4	0.12	47.6 ± 37.9	69.2 ± 131.6	0.048
Lipase at 12 h (*n* = 331)	68.8 ± 224.0	69.4 ± 174.2	0.59	67.5 ± 224.2	71.9 ± 135.1	0.84
Lipase at 24 h (*n* = 328)	45.3 ± 88.2	101.9 ± 256.6	<0.001	58.8 ± 161.9	113.9 ± 266.7	0.001
Maximum Lipase	86.9 ± 219.7	121.1 ± 255.9	0.098	90.8 ± 224.4	134.1 ± 267.3	0.045
**Elevated pancreatic enzyme levels**						
None	105 (77.2)	84 (38.4)		141 (65.9)	48 (34.0)	
Amylase alone	22 (16.2)	105 (47.9)	<0.001 ^a^	55 (25.7)	72 (51.1)	<0.001 ^a^
Lipase alone	5 (3.7)	8 (3.7)	0.29 ^a^	7 (3.3)	6 (4.3)	0.11 ^a^
Both	4 (2.9)	22 (10.0)	<0.001 ^a^	11 (5.1)	15 (10.6)	0.001 ^a^
Post-CA shock	97 (71.3)	188 (85.8)	0.001	157 (73.4)	128 (90.8)	<0.001
CRRT	8 (5.9)	63 (28.8)	<0.001	22 (10.3)	49 (34.8)	<0.001
ECMO	13 (9.6)	26 (11.9)	0.5	18 (8.4)	21 (14.9)	0.06

Data are expressed as mean ± standard deviation or *n* (%); ^a^ frequency is reported in comparison with non-elevated pancreatic enzyme levels. Abbreviations: CA, cardiac arrest; CPR, cardiopulmonary resuscitation; VF, ventricular fibrillation; VT, ventricular tachycardia; ROSC, return of spontaneous circulation; CRRT, continuous renal replacement therapy; ECMO, extracorporeal membrane oxygenation.

**Table 4:** Factors associated with unfavorable neurologic outcomes and 28-day mortality in the multivariable analysis.

	CPC			Mortality		
	OR	95% CI	*p*-Value	OR	95% CI	*p*-Value
Sex, male	2.96	1.20–7.28	0.02			
Witnessed				0.51	0.28–0.91	0.02
Bystander	0.39	0.19–0.81	0.01			
Shockable rhythm (VF/VT)	0.44	0.22–0.85	0.01	0.48	0.25–0.94	0.03
Time from collapse to ROSC (per minute)	1.05	1.04–1.07	<0.001	1.04	1.02–1.05	<0.001
Cardiac cause	0.26	0.12–0.53	<0.001			
**Elevated pancreatic enzyme levels**						
Amylase alone	6.03	3.03–12.00	<0.001 ^a^	2.8	1.58–4.93	<0.001 ^a^
Lipase alone	0.56	0.09–3.37	0.52 ^a^	1.05	0.24–4.56	0.94 ^a^
Both	7.08	1.73–29.02	0.007 ^a^	2.05	0.73–5.77	0.17 ^a^
Post-CA shock				2.78	1.12–6.92	0.03
CRRT	3.77	1.22–11.65	0.02	4.05	1.69–9.72	0.002

^a^ OR is reported in comparison with nonelevated pancreatic enzyme levels. Abbreviations: CPC, cerebral performance category; OR, odds ratio; CI, confidence interval; VF, ventricular fibrillation; VT, ventricular tachycardia; ROSC, return of spontaneous circulation; CA, cardiac arrest; CRRT, continuous renal replacement therapy.

## Data Availability

Datasets used or analyzed during the current study are available from the corresponding author on reasonable request.
